# Supplementary material for: Transcriptome Profiling of Rhipicephalus annulatus Reveals Differential Gene Expression of Metabolic Detoxifying Enzymes in Response to Acaricide Treatment
Source: Biomedicines. 2023 May 6;11(5):1369. doi: 10.3390/biomedicines11051369 (PMC10216723; doi:10.3390/biomedicines11051369)
Supplement: Supplementary file 1 [file biomedicines-11-01369-s001.zip › biomedicines-2175411-supplementary.pdf]

Table S1: Larval packet test for acaricide amitraz against *R.(B) annulatus* tick larvae

Con. Of Acaricide(ppm)	Replicates	Live	Dead	Total	Percentage mortality (%)
Control	R1	42	0	42	0
	R2	49	0	49	0
	R3	89	0	89	0
300	R1	0	84	84	100
	R2	0	60	60	100
	R3	0	52	52	100
150	R1	0	50	50	100
	R2	0	62	62	100
	R3	0	84	84	100
75	R1	2	62	64	96.87
	R2	2	84	90	98.77
	R3	1	62	73	98.63
37.5	R1	52	88	182	71.40
	R2	20	72	72	72.20
	R3	21	130	71	70.40
18.75	R1	30	52	92	67.39
	R2	28	50	93	69.89
	R3	29	62	93	68.81
9.375	R1	22	65	63	65.07
	R2	33	64	93	64.50
	R3	42	41	116	62.06
4.6875	R1	21	60	55	61.81
	R2	19	74	51	62.74
	R3	28	34	80	65.00
2.3437	R1	114	32	151	24.50
	R2	95	52	126	24.60
	R3	71	37	91	21.97
1.171	R1	75	31	100	25.00
	R2	68	20	88	22.70
	R3	72	25	90	20.00
0.585	R1	72	20	94	23.40
	R2	92	18	121	23.90
	R3	46	22	58	20.68
0.292	R1	125	12	137	8.75
	R2	92	10	102	9.80
	R3	110	13	123	10.56
0.146	R1	101	8	109	7.33
	R2	122	10	132	7.57
	R3	93	7	100	7.00

Table S2: Larval packet test for acaricide Flumethrin against *R.(B) annulatus* tick larvae

Con. Of Acaricide (ppm)	Replicates	Live	Dead	Total	Percentage mortality (%)
Control	R1	42	0	42	0
	R2	49	0	49	0
	R3	89	0	89	0
25	R1	0	72	72	100
	R2	0	82	82	100
	R3	0	60	60	100
12.5	R1	0	65	65	100
	R2	0	98	98	100
	R3	0	86	86	100
6.25	R1	0	68	68	100
	R2	0	63	63	100
	R3	0	72	72	100
3.125	R1	0	110	110	100
	R2	0	92	92	100
	R3	0	68	68	100
1.5625	R1	0	88	88	100
	R2	0	52	52	100
	R3	0	71	71	100
0.78125	R1	5	82	87	94.25
	R2	3	60	63	95.24
	R3	3	58	61	95.08
0.39062	R1	4	58	62	93.55
	R2	7	77	84	91.67
	R3	6	79	85	92.94
0.195	R1	5	72	77	93.51
	R2	8	88	96	91.67
	R3	7	76	83	91.57
0.0488	R1	61	47	108	43.52
	R2	43	38	81	46.91
	R3	59	51	110	46.36
0.0122	R1	67	48	115	41.74
	R2	92	71	163	43.56
	R3	104	77	181	42.54

Table S3: Adult Immersion Test data showing the effects of Amitraz against *R.(B) annulatus*

Sl. No	Acaricide	Mean ticks weight per replicate ± SEM (g)	Mean % adult mortality within 15 days ± SEM	Mean eggs mass per replicate ± SEM (g)	Index of fecundity ± SEM	Percentage Inhibition of Fecundity (%)	Hatching % (Visual)
1.	Methanol (control)	08737 ± 0.0391[b]	0 ± 0[a]	0.3947 ± 0.0217[b]	0.4529 ± 0.0224[b]	0	100
2.	200ppm	0.7479± 0.0246[a]	12.4975± 7.9975[a]	0.0438± 0.0052[a]	00.0593± 0.0088[a]	86.91	10
3.	250ppm	0.7670± 0.0296[ab]	12.4950 ± 4.1650[a]	0.0917 ± 0.0232[a]	0.1208 ± 0.0305[a]	73.33	25
4.	300ppm	0.8161 ± 0.0274[ab]	16.6625 ± 6.8035[ab]	0.0882 ± 0.0131[a]	0.1096± 0.0183 [a]	75.80	50
5	350ppm	0.7531 ± 0.0471[a]	33.33 ± 6.8055[ab]	0.0950± 0.0173[a]	0.1271± 0.0251[a]	71.94	50

Table S4: Adult Immersion Test data showing the effects of flumethrin against *R.(B) annulatus*

Sl. No	Acaricide	Mean ticks weight per replicate ± SEM (g)	Mean % adult mortality within 15 days ± SEM	Mean eggs mass per replicate ± SEM (g)	Index of fecundity ± SEM	Percentage Inhibition of Fecundity (%)	Hatching % (Visual)
1.	Methanol (control)	0.845 ± 0.069[a]	0 ± 0[a]	0.425 ± 0.043[b]	0.501 ± 0.017[c]	0	100
2.	20 ppm	0.882± 0.065[a]	4.165 ± 4.16[a]	0.0508 ± 0.017[a]	0.053 ± 0.018[b]	89.22	5
3.	30 ppm	0.910 ± 0.039[a]	8.33 ± 4.809[ab]	0 ± 0[a]	0 ± 0[a]	100	0
4.	40 ppm	0.895± 0.040[a]	8.33 ± 4.809[ab]	0 ± 0[a]	0 ± 0[a]	100	0
5	60 ppm	0.891 ± 0.020[a]	8.33 ± 4.809[ab]	0 ± 0[a]	0 ± 0[a]	100	0
6	80 ppm	0.919± 0.043[a]	12.49 ± 4.165[ab]	0 ± 0[a]	0 ± 0[a]	100	0
7	100 ppm	0.877 ± 0.052[a]	20.82 ± 4.167[b]	0 ± 0[a]	0 ± 0[a]	100	0

Table S5: De-novo Assembly Statistics of the untreated and treated tick samples

**Sample Name**	**Untreated**	**Treated**
Tool used	Trinity	
Hash length	25	
Transcripts Generated:	76491	52471
Maximum Contig Length:	15075	15881
Minimum Contig Length:	201	201
Average Contig Length:	702.8 ± 906.6	560.2 ± 647.3
Median Contig Length:	996	979
Total Transcripts Length:	53760902	29393110
Total Number of Non-ATGC Characters:	0	0
Percentage of Non-ATGC Characters:	0	0
Transcripts >= 100 bp:	76491	52471
Transcripts >= 200 bp:	76491	52471
Transcripts >= 500 bp:	27658	15790
Transcripts >= 1 Kbp:	13435	6101
Transcripts >= 10 Kbp:	28	4
Transcripts >= 1 Mbp:	0	0
N50 value:	1169	731

Table S6: Statistics of SSR Analysis from *R.(B) annulatus* Transcriptome

Total number of sequences examined:	92341
Total size of examined sequences (bp):	58009214
Total number of identified SSRs:	18749
Number of SSR-containing sequences	13755
Number of sequences containing more than 1 SSR:	3199
Number of compound SSRs	1195
Mononucleotide Repeats (p1)	11769
Di nucleotide Repeats (p2)	2337
Tri nucleotide Repeats (p3)	1179
Tetra nucleotide Repeats (p4)	3457
Pentanucleotide Repeats (p5)	7
Hexa nucleotide Repeats (p6)	0

Table S7: Primers used to analyze transcript levels of selected CYP and GST from transcriptome data in *R.(B)annulatus*

**Unigene ID**	**Transcript ID**	**Forward Primer**	**Reverse primer**
Master_Control_80954	treated30980_g1_i1(CYP450)	TCCAGTTCACGCACAAAGAC	CATATTGCTTGGCCCATTTC
Master_Control_78390	treated27576_g2_i1(CYP450)	GGCTGACTTGTTCGCTATGC	CAGGAGAACACCACACCTCA
Master_Control_23876	Untreated_c38633_g1_i1(CYP450)	AGGTTCATCGGTGCCAGA	GGCCAGCTGGTTTATCCAC
Master_Control_48389	Untreated_c51361_g2_i1(CYP450)	TCGAGATTTCATTGACGGATAC	TTGAGTGTGGGTTGTTTTCG
Master_Control_20970	Untreated_c36267_g1_i1(CYP450)	TCGATTTTACGACGCTACCC	TTTCCTCGACAGCTTCTGCT
Master_Control_40516	Untreated_c48268_g3_i1(CYP450)	CAGATCAGGCTCCGCAAC	CGCCTATTTGGGTCCTTTG
Master_Control_89008	treated41222_g1_i1(CYP450)	AGCATCTCGCTCTCTGTCGT	CCAAGATGTGCAACAGAAGG
Master_Control_57282	Untreated_c55171_g1_i1(GST)	CGCAACATCACTCGTGGTT	CTCCGAAGCAGTACTTTCCAG
Master_Control_84052	treated34065_g1_i1(GST)	TTTGAGCGCGAACTTTATCA	CGCGAATGTCCCAGTAGC
	Beta Actin	AAGGACCTGTACGCCAACAC	GGCGATACCGGGGTACAT
